# Metastatic Rectal Cancer in a 52-Year-Old Woman: Advocating for Proactive Screening

**DOI:** 10.7759/cureus.66852

**Published:** 2024-08-14

**Authors:** Karelis Lopez Muniz, Denver J Stutler, Marianna Zeichen

**Affiliations:** 1 School of Medicine, St. George's University, St. George's, GRD; 2 Colorectal Surgery, Jackson Memorial Hospital, Miami, USA

**Keywords:** colonoscopy, lung nodule, rectal mass, metastatic rectal cancer, asthma

## Abstract

We present a case of metastatic rectal cancer in a 52-year-old woman, initially manifested as an acute asthma exacerbation. The patient was referred to the colorectal surgery team due to the discovery of a rectal mass after she sought treatment for shortness of breath following Clorox (cleaning product) exposure. Imaging revealed a right upper lobe nodule alongside a significant rectal mass, leading to a diagnosis of stage 4 colorectal cancer via colonoscopy and flexible sigmoidoscopy. The rectal mass was subsequently resected through robotic laparoscopic low anterior resection with coloproctostomy. This case underscores the need for clinicians to consider atypical presentations of colorectal cancer, such as asthma exacerbation, particularly in the context of the rising incidence of early-onset colorectal cancer (eoCRC). The survival rates for localized colorectal cancer are markedly higher than for metastatic cases, amplifying the need for timely and completed colorectal cancer screening. This report emphasizes the importance of increased awareness, timely diagnosis, and personalized screening strategies to improve outcomes in younger patients with colorectal cancer.

## Introduction

Colorectal cancer (CRC) is a significant health concern globally, with rising incidence rates observed among younger individuals. A recent study in the American Cancer Society found that the proportion of cases among individuals aged 20-54 years increased from 11% in 1995 to 20% in 2019 [1]. Most patients with rectal cancer complain of symptoms such as rectal bleeding, positive fecal occult blood testing (FOBT), anemia, lower abdominal pain, and constipation [2].

This case underscores the pressing need to address the rising incidence of early-onset colorectal cancer (eoCRC). Research indicates that the proportion of cases among individuals aged 20-54 years has increased dramatically over recent decades, emphasizing the necessity for heightened awareness and proactive screening strategies [3]. As the five-year survival rate for localized CRC remains markedly higher than for metastatic cases, timely diagnosis and intervention are critical. Research has found that the stage at diagnosis is the most important predictor of survival, with five-year relative survival ranging from 91% for localized disease to 14% for distant disease [1].

Moreover, the complexities surrounding CRC screening adherence among younger populations pose ongoing challenges. This report not only highlights a unique clinical presentation but also serves as a call to action for healthcare providers to remain vigilant in recognizing atypical manifestations of colorectal cancer, particularly in the context of rising eoCRC rates. By fostering interdisciplinary collaboration and implementing personalized screening protocols, we can aim to improve outcomes and mitigate the burden of colorectal cancer on our healthcare system.

## Case presentation

This case report highlights the emergence of early-onset colorectal cancer (eoCRC) as a distinct clinical entity, exemplified by the case of a 52-year-old woman presenting with metastatic rectal cancer. Her clinical journey began with an acute episode of shortness of breath following exposure to Clorox (a cleaning product), which ultimately led to the discovery of a pulmonary nodule and a rectal mass. The patient had a past medical history notable for asthma, which was under control at that moment with a rescue inhaler. She suffered from glaucoma, which was stable at the moment and in no need for surgery. She also had sickle cell trait with no associated symptoms and no treatment. She exhibited no typical symptoms associated with rectal cancer, such as weight loss, constipation, rectal bleeding, or abdominal distension. Imaging studies revealed concerning findings, including a spiculated pulmonary nodule and a significant rectal mass, prompting further investigation. Subsequent colonoscopy confirmed the presence of a large friable mass and polyps, with pathology revealing high-grade dysplasia.

The patient was referred to the colorectal surgery service by oncology due to a rectal mass. The patient did not undergo any colonoscopies in the past. Chest X-ray showed incidental finding of right upper lobe 12 mm nodule. A computer tomography scan without contrast showed a 12 mm spiculated nodule and a 12 nodule on the right lung. Fluorodeoxyglucose (FDG)-positron emission tomography (PET) scan showed no avid chest wall lesions, no mediastinal lymph nodes, and no other FDG avid pulmonary nodules. There was a right lateral rectal mass measuring 2.2 x 2.3 cm, and there were small left pelvic sidewall sub-centimeter nodules but with accompanying increased FDG activity. A colonoscopy was then performed, which showed a large friable mass in her rectum, which was 12 cm from the anal verge, a tattoo was placed, and 2 pedunculated polyps in the sigmoid colon were removed. Pathology showed a tubular adenoma from the sigmoid, tubulovillous adenoma with high-grade dysplasia in the rectum. The patient was then discharged and referred to oncology. 

**Figure 1:**
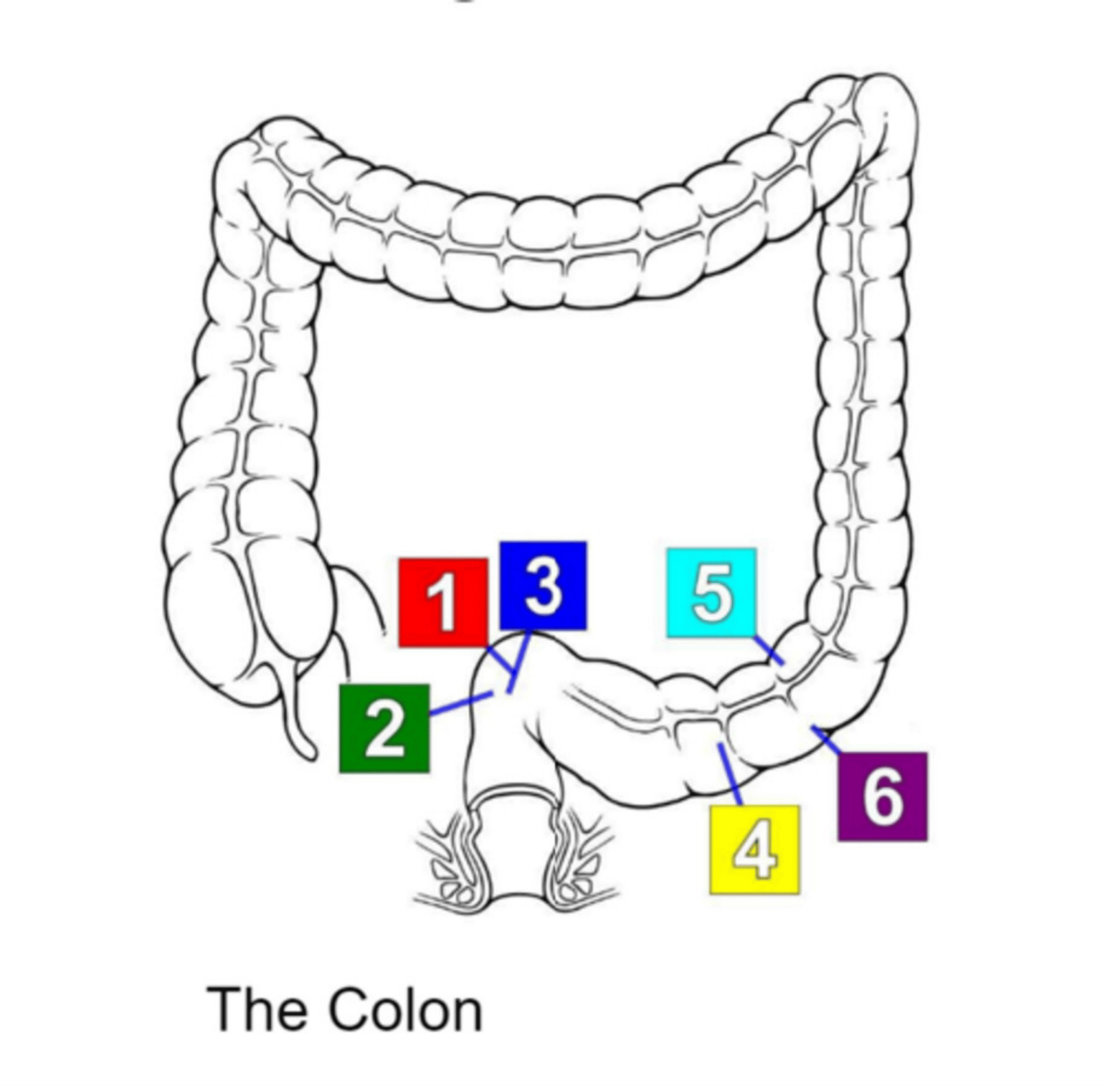
Diagram indicating anatomical locations of pathological images taken. Please refer to Figure 2 for the pathological images taken. 1-3: Superior rectum; 4-6: Sigmoid colon. Image credit: Marianna Zeichen. Permission was granted for use in this article.

**Figure 2:**
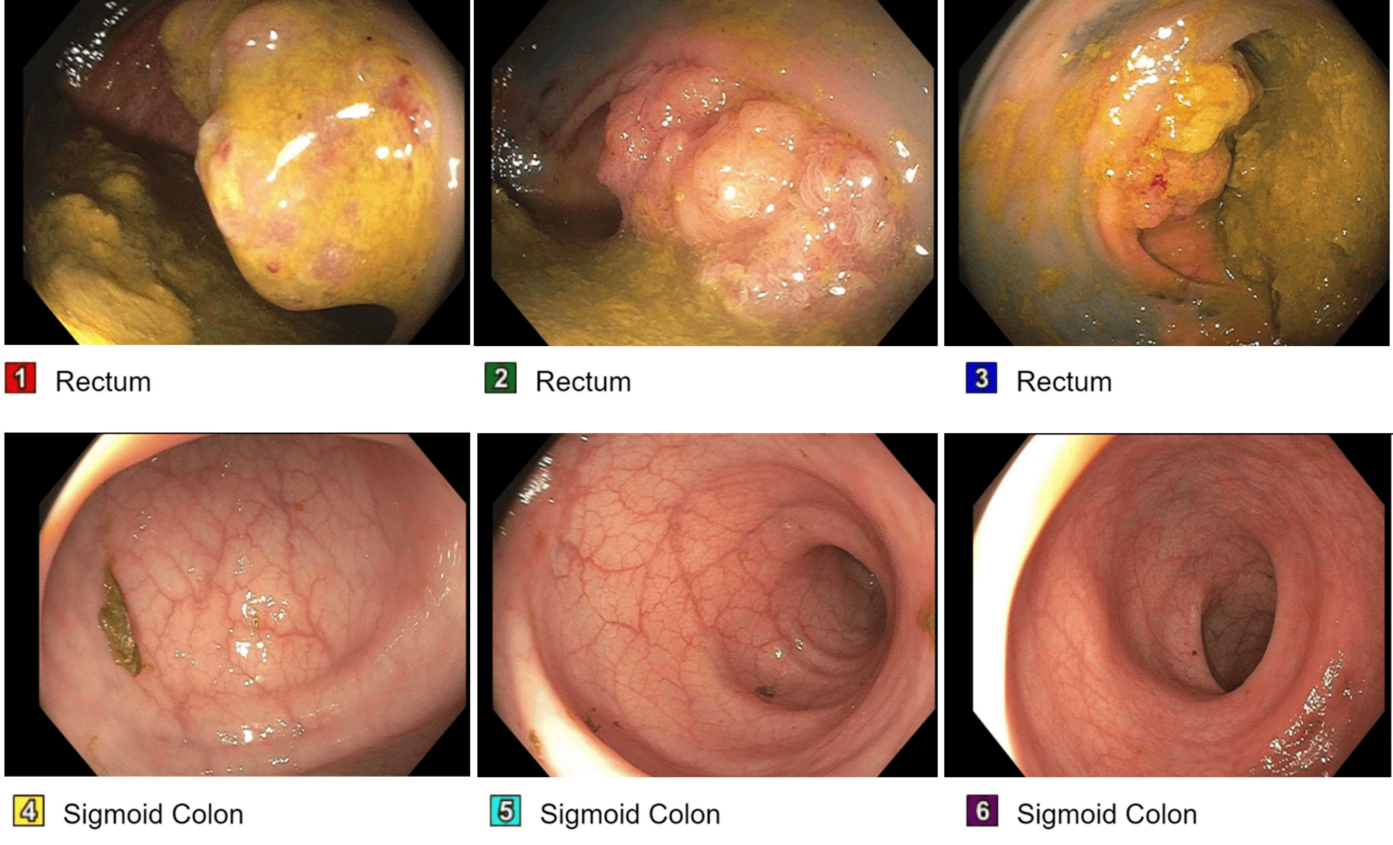
Images of the colon and rectum taken during flexible sigmoidoscopy. Please refer to Figure 1 for the anatomical location of the images. Images 1-3 were taken of the superior rectum; note the frond-like/villous non-obstructing medium-sized mass found during sigmoidoscopy. Images 4-6 were taken in the sigmoid colon; note the full extent of the exam which found no further abnormalities.

Oncology referred the case to colorectal surgery for possible rectal mass resection. Pre-operative flexible sigmoidoscopy was performed, which showed a non-circumferential frond-like/villous non-obstructing medium-sized mass in the rectum (Figures 1, 2). Biopsies were taken with cold large-capacity forceps, which showed fragments of tubulovillous adenoma with multifocal high-grade dysplasia/intramucosal adenocarcinoma.

**Figure 3:**
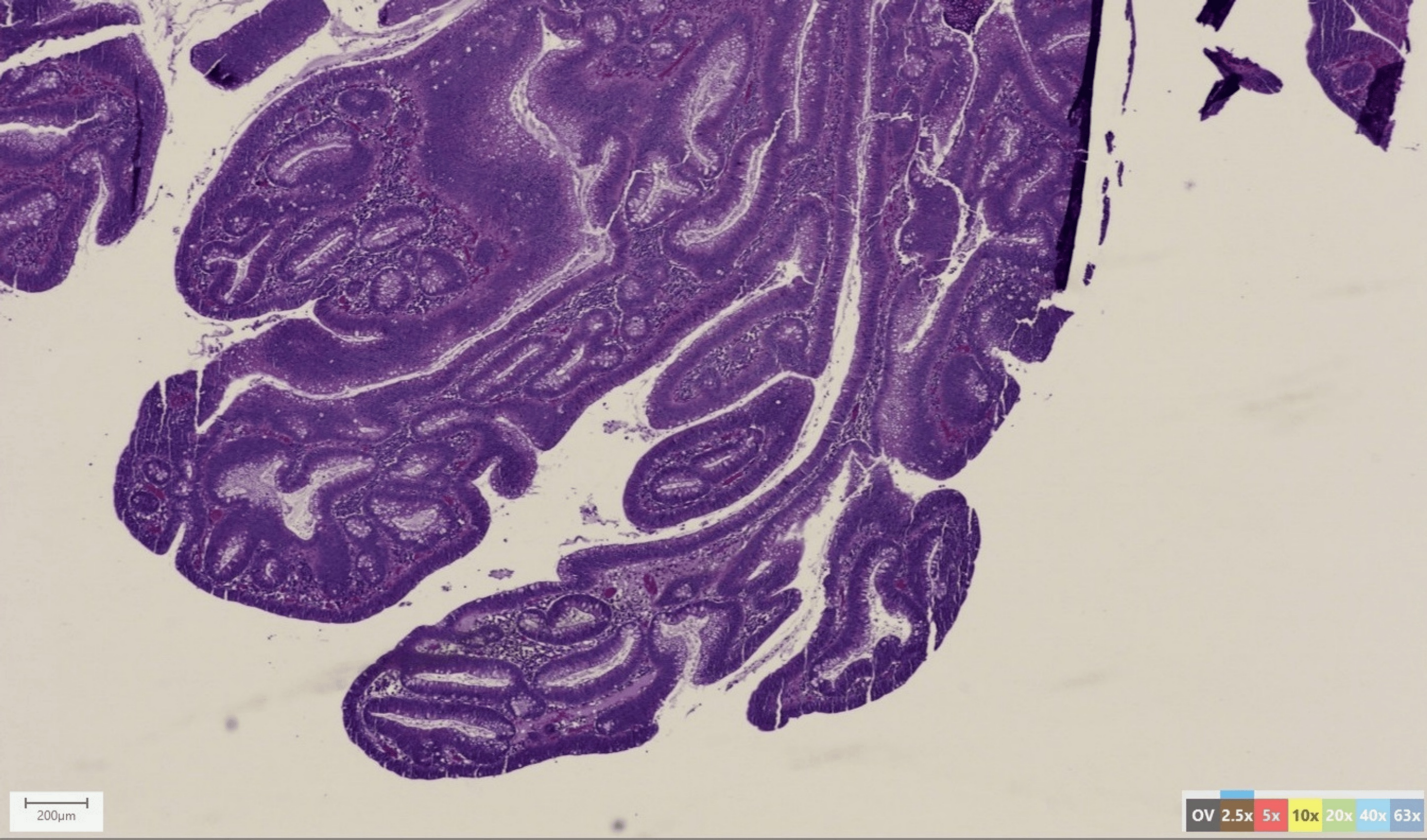
Histologic image of the resected specimen. Histopathology of tissue biopsy at 2.5 magnification stained with hematoxylin & eosin.

**Figure 4:**
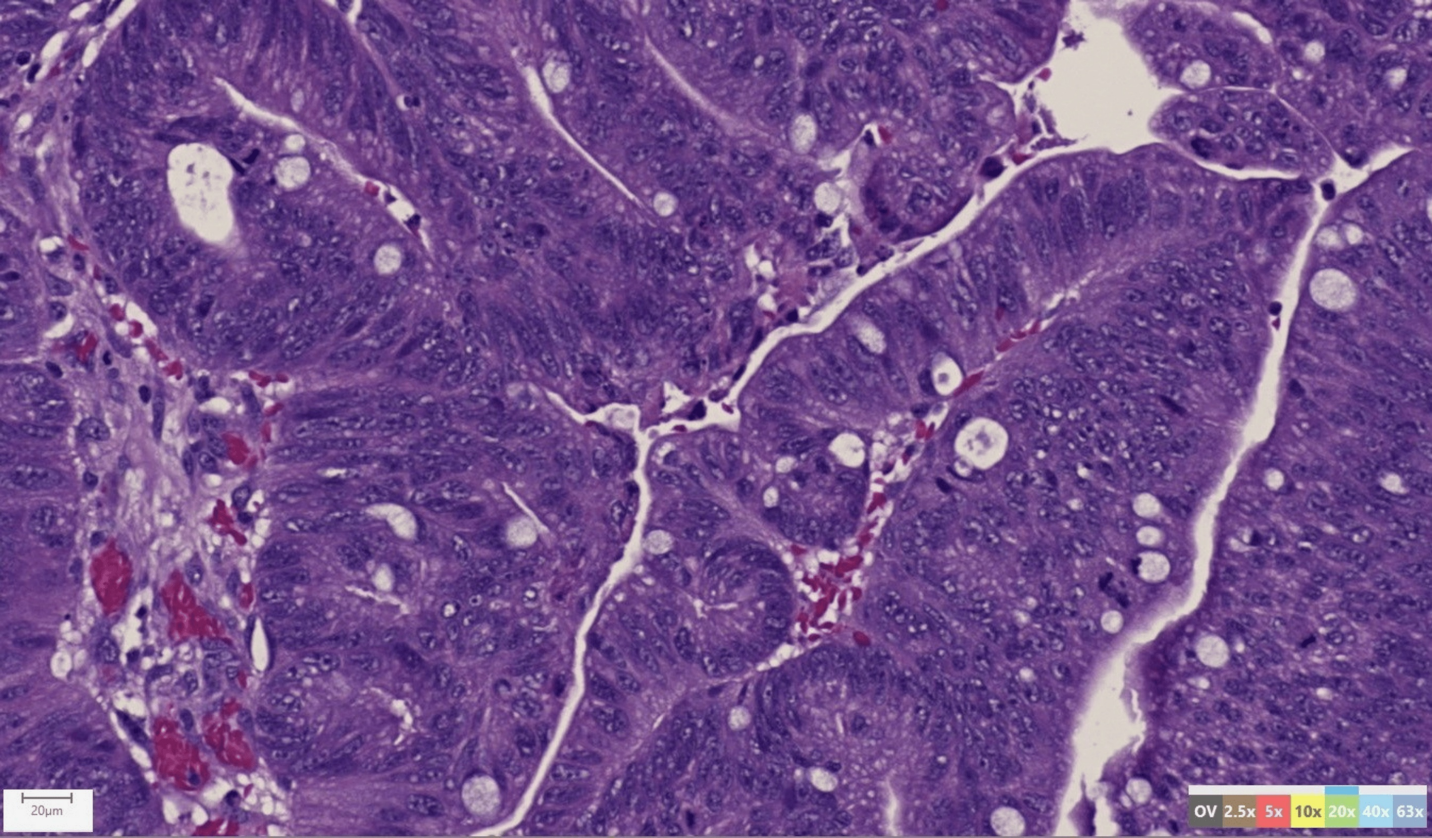
Histologic image of the resected specimen. Histopathology of tissue biopsy at 20 magnification stained with hematoxylin & eosin.

The patient then underwent an uncomplicated robotic lower anterior resection (LAR) with coloproctostomy. The surgical pathology report showed fragments of tubulovillous adenoma with multifocal high-grade dysplasia/intramucosal adenocarcinoma (Figures 3, 4).

The patient was seen in the outpatient clinic for a post-op visit. The patient followed up with the colorectal surgical team within two weeks of surgery, and plans to undergo a colonoscopy in one year and then again in three years. At that time, she was doing well, with no issues, passing gas, having bowel movements, and tolerating diet. She is currently pending a surveillance colonoscopy in one year and plans to follow up with oncology.

## Discussion

In this report, we bring to attention a rare presentation of metastatic colorectal cancer.

The case of metastatic rectal cancer in a 52-year-old woman initiates the conversation on several key aspects of early-onset colorectal cancer (eoCRC). It was found by Adigun et al. that the incidence of eoCRC has steadily increased over the past few decades, with younger individuals representing a significant proportion of newly diagnosed cases [3].

It was found in an American Cancer Society study that the proportion of cases among individuals aged 20-54 years increased from 11% in 1995 to 20% in 2019 [1]. The study also identified that between 2011 and 2020, colon cancer mortality among individuals below 50 years increased by 0.5%-3% annually [1].

Moreover, the importance of timely detection cannot be overstated, as evidenced by this patient's presentation with metastatic disease. According to the American Cancer Society, the five-year survival rate for localized CRC is approximately 90%, whereas the survival rate drops to around 14% for metastatic disease [4]. However, challenges persist in achieving optimal CRC screening uptake and adherence, particularly among younger populations.

Future studies should focus on personalizing the screening process according to an individual's risk for CRC, as recommended by Adigun et al. [3]. Risk-stratified screening protocols, tailored to specific genetic predispositions, family history, and lifestyle factors, hold promise in optimizing the allocation of screening resources and improving the effectiveness of CRC screening programs.

In the United States of America, CRC screening rates remain suboptimal, with only 67% of patients up to date with screening as of 2021 [5]. Achieving universal screening, as recommended by Cubiella et al., with the participation of primary care in collaboration with hospital care, is crucial for improving screening rates and reducing the burden of CRC [6].

Another study, conducted at a population level, examined the potential impact of adherence to current screening guidelines on preventing eoCRC. Their findings indicated that strict adherence could have accelerated the detection of 52% of eoCRC cases and possibly averted 16% [7]. To address the early diagnosis of early-onset colorectal cancer (eoCRC) among individuals aged 40 to 49 years, researchers explored various approaches. Gupta et al. noted that a significant proportion of eoCRC cases in this age bracket qualified for early screening due to familial history [8]. These results emphasize the need for good screening protocols as well as family history taking to identify high-risk patients.

Notably, having a first-degree relative diagnosed with colorectal cancer doubles one's risk of developing the disease. Thus, the US Multisociety Task Force on Colorectal Cancer (USMSTF) recommends initiating colonoscopy screening no later than age 40 for high-risk individuals, with the option to begin earlier based on familial history, aiming for screening to commence 10 years prior to the age at which the relative was diagnosed [8].

However, challenges remain in achieving optimal CRC screening rates, particularly among younger populations. Despite public health messaging endorsing age-based screening, individuals under age 50 are often overlooked in screening initiatives. Hyams et al. emphasize the importance of understanding the attitudes and barriers to screening among this population, suggesting the need for tailored interventions to address their unique needs and preferences [9].

Embracing interdisciplinary collaboration and evidence-based practices, as highlighted by Álvarez-Delgado et al., is essential in navigating the complexities of eoCRC diagnosis and treatment [10]. Collaborative decision-making involving patients, healthcare practitioners, and multidisciplinary teams can enhance the quality of care and improve patient outcomes.

Data from a questionnaire completed by Americans in November 2019 found that the top barrier to people with no prior screening was a lack of knowledge [11]. In addition, the top barrier to obtaining multi-target stool DNA (mt-sDNA) and guaiac fecal occult blood test (gFOBT) was a lack of provider recommendation [11]. The same data collected found that psychosocial barriers (e.g., too painful, unpleasant, or embarrassing; worry or fear about positive results) were the most commonly reported obstacles to colonoscopy. This information necessitates the crucial role that primary care providers place in population screening rates. 

In summary, the case of metastatic rectal cancer in a 52-year-old woman underscores the urgent need for a concerted effort to address early-onset colorectal cancer. Through collaborative efforts, innovative approaches, and targeted interventions, healthcare systems can take the steps necessary to effectively manage eoCRC and strive towards better outcomes and improved overall population health.

## Conclusions

This case report of a patient with metastatic rectal cancer presenting as an acute asthma exacerbation highlights the critical need for increased awareness and vigilance among healthcare providers regarding the atypical manifestations of colorectal cancer, particularly in younger populations. The rising incidence of early-onset colorectal cancer (eoCRC) demands proactive screening strategies and personalized approaches to identify high-risk individuals effectively. Given the stark contrast in survival rates between localized and metastatic disease, timely diagnosis and intervention are essential for improving patient outcomes. By fostering interdisciplinary collaboration and addressing barriers to screening, healthcare systems can better manage eoCRC and enhance overall population health, ultimately reducing the burden of colorectal cancer.

## References

[1] Siegel RL, Wagle NS, Cercek A, Smith RA, Jemal A (2023). Colorectal cancer statistics, 2023. CA Cancer J Clin.

[2] Holtedahl K, Borgquist L, Donker GA (2021). Symptoms and signs of colorectal cancer, with differences between proximal and distal colon cancer: a prospective cohort study of diagnostic accuracy in primary care. BMC Fam Pract.

[3] Adigun AO, Adebile TM, Okoye C, Ogundipe TI, Ajekigbe OR, Mbaezue RN, Okobi OE (2023). Causes and prevention of early-onset colorectal cancer. Cureus.

[4] Chetroiu D, Pop CS, Filip PV, Beuran M (2021). How and why do we screen for colorectal cancer?. J Med Life.

[5] Sabatino SA, White MC, Thompson TD, Klabunde CN (2015). Cancer screening test use - United States, 2013. MMWR Morb Mortal Wkly Rep.

[6] Cubiella J, Marzo-Castillejo M, Mascort-Roca JJ (2018). Clinical practice guideline. Diagnosis and prevention of colorectal cancer. 2018 update. Gastroenterol Hepatol.

[7] Stanich PP, Pelstring KR, Hampel H, Pearlman R (2021). A high percentage of early-age onset colorectal cancer is potentially preventable. Gastroenterology.

[8] Gupta S, Bharti B, Ahnen DJ (2020). Potential impact of family history-based screening guidelines on the detection of early-onset colorectal cancer. Cancer.

[9] Hyams T, Mueller N, Curbow B, King-Marshall E, Sultan S (2022). Screening for colorectal cancer in people ages 45-49: research gaps, challenges and future directions for research and practice. Transl Behav Med.

[10] Álvarez-Delgado A, García ML, García-González JM (2021). Improvements in the effectiveness of early detection in colorectal cancer with open-label randomised study. J Clin Med.

[11] Zhu X, Parks PD, Weiser E, Jacobson DJ, Limburg PJ, Finney Rutten LJ (2021). Barriers to utilization of three colorectal cancer screening options - data from a national survey. Prev Med Rep.

